# A Systematic Solution for Moving-Target Detection and Tracking While Only Using a Monocular Camera

**DOI:** 10.3390/s23104862

**Published:** 2023-05-18

**Authors:** Shun Wang, Sheng Xu, Zhihao Ma, Dashuai Wang, Weimin Li

**Affiliations:** 1 Guangdong Provincial Key Lab of Robotics and Intelligent System, Shenzhen Institute of Advanced Technology (SIAT), Chinese Academy of Sciences (CAS), Shenzhen 518055, China; 2 Shandong Institute of Advanced Technology, CAS, Jinan 250102, China; 3 School of Control Science and Engineering, Shandong University, Jinan 250061, China; 4 School of Microelectronics, Southern University of Science and Technology, Shenzhen 518055, China

**Keywords:** moving-target detection, optical flow, monocular vision, 3D target tracking, cubature Kalman filter

## Abstract

This paper focuses on moving-target detection and tracking in a three-dimensional (3D) space, and proposes a visual target tracking system only using a two-dimensional (2D) camera. To quickly detect moving targets, an improved optical flow method with detailed modifications in the pyramid, warping, and cost volume network (PWC-Net) is applied. Meanwhile, a clustering algorithm is used to accurately extract the moving target from a noisy background. Then, the target position is estimated using a proposed geometrical pinhole imaging algorithm and cubature Kalman filter (CKF). Specifically, the camera’s installation position and inner parameters are applied to calculate the azimuth, elevation angles, and depth of the target while only using 2D measurements. The proposed geometrical solution has a simple structure and fast computational speed. Different simulations and experiments verify the effectiveness of the proposed method.

## 1. Introduction

Moving-target detection and tracking is widely required by and applied to various applications, such as security systems, obstacle detection, and search-and-rescue missions [1,2,3,4]. Consequently, the research topic of moving-target detection and tracking has attracted much attention. Previous studies about moving-target detection and tracking can be divided into different methods according to the applied sensors, e.g., lidar-, sonar-, somatosensory-, and visual-based methods [5,6]. The lidar-based method is mainly used for indoor applications, and a certain number of reflective plates need to be installed in the environment. In addition, the installation accuracy and installation location have high requirements; thus, costs are high. The sonar-based method is always used for underwater scenarios. The somatosensory-based method requires some special sensors to be carried by the targets, which are not available, especially in security applications. Compared with the above transmission methods, the visual-based method is the most popular in civilian applications. Due to the rapid development of camera sensors, the visual-based method has many advantages, e.g., low cost, a high signal-to-noise ratio, convenient transmission, and fast operation speed [7,8]. Therefore, it is an appropriate choice for moving-target detection and tracking, even for microtargets [9,10,11].

In computer vision, moving-target detection has two steps, i.e., removing redundant information, and extracting the region with positional changes in the image sequence. In the 1970s, Jain R. et al. proposed a method to detect moving targets by using scene changes between adjacent frames called the frame difference method [12]. The main advantage of the frame difference method is the low computational complexity. Thus, it is simple to implement the algorithm, and it can adapt to different dynamic environments with various lighting conditions. However, it cannot detect all the pixels of a moving target [13], and the accuracy of the detection result is limited [14]. In [15], the traditional frame difference method was improved with a three-frame difference method to calculate the difference. A moving-vehicle video detection method that combines ViBe and frame difference was proposed in [8]. It improves the background update efficiency and can detect moving vehicles in the video. The background difference method developed in [16] has low complexity. However, it has the “tailing” problem and requires constant illumination. In [17], the difficulty of the frame difference, i.e., background modeling and real-time updating of subsequent models, was overcome. The authors of [18] proposed a moving-object detection method through the brief background subtraction technique. In [19], an intelligent background differential model was designed for training target monitoring, and a background difference method based on RGB color separation was proposed.

The optical flow technique is an effective, widely applied method. In 1981, Horn and Schunck first connected an image’s grayscale with the optical flow vector and proposed the basic model of optical flow calculation [20]. Subsequently, on the basis of this model, various methods for optical flow calculation have been developed. The Lucas–Kanade optical flow method is a widely used difference algorithm for optical flow calculation. However, the Lucas–Kanade optical flow method needs to simultaneously meet three assumptions, so it is challenging to meet these strong constraints [21]. The pyramid optical flow method was created to compensate for the Lucas–Kanade optical flow method [22]. With the development and successful application of deep-learning technology in images [23], many computing methods have been developed by introducing deep learning into the field of optical flow estimation. In 2016, SpyNet was able to estimate the motion between two images by using only a small network with the help of a spatial pyramid and image mapping [24]. PWC-Net was proposed in 2018 to construct the image feature pyramid on the basis of the traditional pyramid optical flow method. At the same time, the number of network parameters is significantly reduced to about 0.06 times that of FlowNet2.0 [25]. In 2018, lightweight optical flow estimation network LiteFlownet was proposed [26]. MaskFlownet was proposed in 2020 [27]. Compared with PWC-Net, it effectively overcomes the ghosting problem and improves detection accuracy. Some solutions using artificial intelligent algorithms were also proposed at the expense of computational complexity [28,29].

According to the number and characteristics of visual sensors, existing location systems can be roughly divided into multi-vision locating, binocular vision locating, and monocular vision locating [30]. Multi-vision can obtain more visual information, but the structure of the visual controller is complex [31], and real-time performance is poor. Binocular vision needs to accurately know the spatial position relationship between the two cameras, collect two images from different perspectives, and then calculate the 3D coordinates of a target according to the pixel difference of the two images through the triangulation principle. Stereo matching is complex [32]. Monocular vision locating only needs to estimate the target orientation and distance information through the inherent performance parameters of the camera, which is low-cost and easy to implement. The system structure is relatively simple and has low requirements for computing resources, so it has been widely used [33]. On the basis of monocular vision, a target azimuth estimation algorithm in range-gated imaging was proposed in [34] representing the target azimuth through the projection relationship between coordinate systems and trigonometric function. A systematic procedure of model-referenced pose estimation was introduced in [35] to obtain the relative pose information between an underwater vehicle and underwater structures whose geometry and shape are known. The authors in [36] proposed an iterative algorithm that could estimate a target’s pose from an image according to the size of the target. On the basis of the geometric model of camera imaging, the camera’s parameters and the principle of an analog signal could be transformed into a digital signal.

Conventional moving target tracking based on visual image information focuses on the 2D image level and cannot perform realizations in a 3D space. However, 2D tracking has certain limitations. It can only obtain motion information from two directions of the moving target. However, the realization of the 3D tracking of moving targets is fundamental in industrial control. In recent years, there have been many visual devices to obtain the 3D spatial information of moving targets, such as the RealSense depth camera, Kinect camera, and Leap Motion somatosensory device [37]. However, such devices are usually expensive, have different limitations, and are always used in academic research fields [38]. Using computer technology and an ordinary optical camera to obtain 3D information at a low cost is greatly significant. The Kalman filter (KF) is the most commonly used tool for the 3D tracking of moving targets. For nonlinear motion, researchers improved the KF and then proposed many suitable methods for nonlinear motion, such as the extended Kalman filter (EKF), unscented Kalman filter (UKF), quadrature Kalman filter (QKF), and cubature Kalman filter (CKF) [39,40,41].

To summarize, there are some solutions for moving-target detection and tracking based on visual images, but the limitations of their complexity and cost have not been fully addressed. Specifically, some key problems are listed below. (1) The noise problem seriously affects the detection effect of moving targets. (2) Most previous papers about visual-based target detection used a 3D camera, which has the disadvantages of inaccurate depth estimation and complex computation. Previous methods only using a monocular camera could hardly satisfy the practical requirements. (3) Systematic solutions, including improvements in target detection and estimation, have not been fully addressed. This paper achieves significant improvements according to our previous work [42]. A systematic method is proposed for mobile-target detection and tracking. The three key contributions of this paper are presented below.

- An improved optical flow method with modifications in the PWC-Net is applied, and the K-means and aggregation clustering algorithms are improved. Thus, a moving target could be quickly detected and accurately extracted from a noisy background.
- A geometrical solution using pinhole imaging theory and the CKF algorithm is proposed that has a simple structure and fast computational speed.
- The proposed method was verified with sufficient simulation and experimental examples, which has significant value for practical applications.

This paper is organized as follows. Section 2 introduces some related previous methods and the problem formulation. The optical flow-based moving target detection and extraction method is developed in Section 3. Section 4 proposes the pinhole geometrical target state estimation algorithm in detail. A CKF algorithm is modified in Section 5 to further improve the estimated results from the geometrical solution. The experimental verification of the proposed method is provided in Section 6. Lastly, Section 7 gives the conclusion.

## 2. Preliminaries

### 2.1. Pyramid Lucas–Kanade Optical Flow Method

The classical optical flow method comprises two parts: obtaining the optical flow basic constraint equation and calculating the optical flow value. To acquire the primary constraint equation of optical flow, two assumptions should be satisfied: (i) the brightness of the same object in different pictures taken at diverse time instants should be uniform, and (ii) the positional change of the moving target between adjacent frames is small. We define the central position of a target in an image as (x,y), and its gray value is I(x,y). The target central point moves to position $\left(x+d_{x},y+d_{y}\right)$ in the next frame after Δt, and the gray value becomes $I(x+d_{x},y+d_{y},t+\Delta t)$. On the basis of the first assumption that the brightness is uniform, the following equation holds,

(1) $$I\left(x,y,t\right)=I\left(x+d_{x},y+d_{y},t+\Delta t\right).$$

On the basis of the second hypothesis, we can acquire the Taylor expansion of (1), which takes the following form:

(2) $$I\left(x,y,t\right)=I\left(x,y,t\right)+\frac{\partial I}{\partial x}d_{x}+\frac{\partial I}{\partial y}d_{y}+\frac{\partial I}{\partial t}\delta t+\varepsilon ,$$

where ε is the second-order infinitesimal term that can be ignored since Δt→0. Therefore, we have

(3) $$\frac{\partial I}{\partial x}d_{x}+\frac{\partial I}{\partial y}d_{y}+\frac{\partial I}{\partial t}\Delta t=0.$$

We define u,v as the velocity component of the optical flow along the coordinate axes *x* and *y*, and $u=\frac{d_{x}}{\Delta t}$, $v=\frac{d_{y}}{\Delta t}$. $I_{x}=\frac{\partial I}{\partial x}d_{x}$, $I_{y}=\frac{\partial I}{\partial y}d_{y}$ and $I_{t}=\frac{\partial I}{\partial t}$ is the partial derivative of the gray value of the image pixel along the x,y,t directions; thus, the following basic constraint equation of optical flow is obtained:

(4) $$I_{x}u+I_{y}v+I_{t}=0.$$

**Figure 1:**
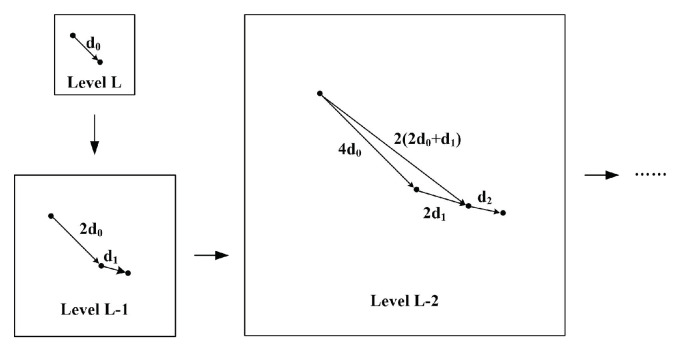
Pyramid Lucas-Kanade optical flow method.

In order to obtain a unique u,v, many calculation methods can be applied. The most popular one is the Lucas–Kanade optical flow method. To use the Lucas–Kanade optical flow method, another assumption should be held as an extra constraint equation of optical flow. The assumption is that the same neighbors’ pixels have the same motion. Specifically, the projected points of neighboring points in the 3D space are also the nearest neighbors in the image. On the basis of this assumption, the system equation of neighborhood pixels can be added, and the optical flow of regional central pixels can be obtained using the weighted least-squares method [42]. The Lucas–Kanade (LK) optical flow method needs to simultaneously meet three assumptions, and the corresponding constraints should be satisfied. Therefore, LK optical flow method cannot be applied directly. Otherwise, the calculated optical flow has large errors. The pyramid LK optical flow method is an appropriate solution [22]. In the pyramid Lucas–Kanade optical flow method as shown in Figure 1, we first need to establish the image pyramid, which includes image smoothing through a Gaussian filter, and sample collection to reduce the image resolution. The image resolution of each layer is reduced to half its value after each layer’s sampling process. Thus, the large displacement motion is gradually reduced and decomposed into several small displacement motions for optical flow accumulation calculation that are suitable for high-speed motion. In the iterative solution, layer by layer, the optical flow is calculated with the LK optical flow method from the top of the image pyramid. Then, the optical flow calculated from the image of Layer L-1 is taken as the initial value of the optical flow estimation of the Layer L-2 image. This process is repeated until the optical flow at the bottom of the image pyramid is calculated. The pyramid operations can satisfy the three assumptions mentioned before, and the optical flow can be calculated accurately. For example, if the maximal displacement of pixel motion processed by the LK optical flow method is $d_{\max}$, the maximal displacement of pixel motion processed by the pyramid LK method becomes $d_{\max final}=\left(2^{L+1}-1\right)d_{\max}$. Thus, the optical flow calculation error of the large-displacement moving target is significantly reduced.

### 2.2. Target Localization Using Monocular Vision

**Figure 2:**
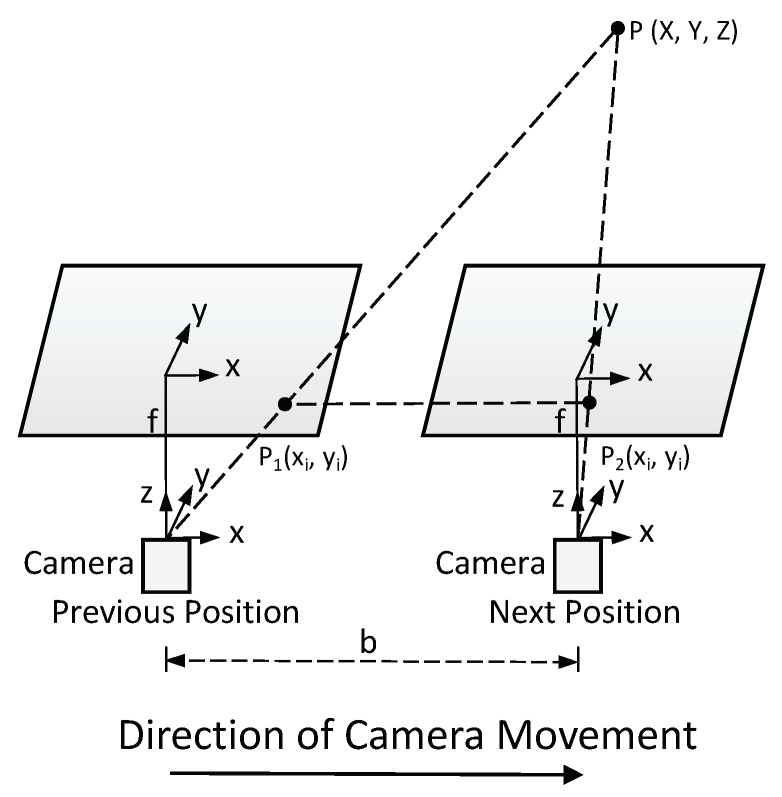
Schematic diagram of monocular visual–spatial target localization.

**Figure 3:**
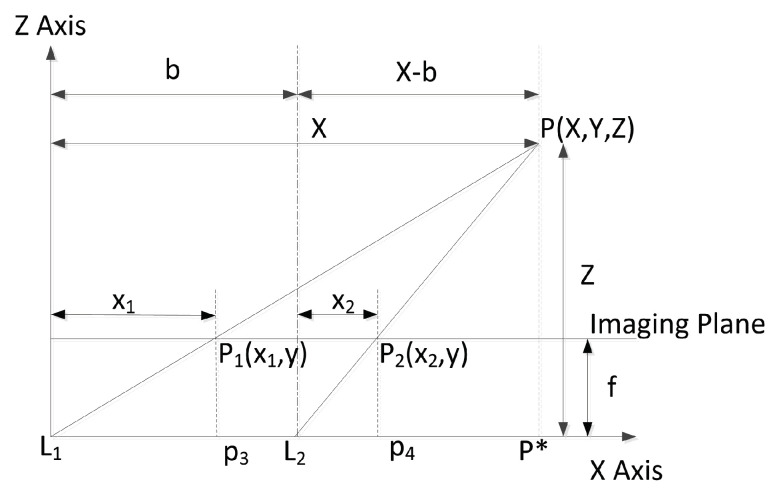
Two-dimensional front view of monocular vision spatial target localization (from the camera side).

Previous 3D spatial localization methods only used monocular cameras and were generally based on processing two images acquired from different positions. An example of locating one target using two images is shown in Figure 2. In order to understand the monocular visual–spatial localization method of two images, we drew a 2D plane schematic diagram, as shown in Figure 3. The positions of the camera’s optical center before and after the translation are $L_{1}$, $L_{2}$, the position of L1 is the origin, the camera optical axis is along the *Z* axis, and the perpendicular direction to the camera optical axis is the *X* axis that established a 2D rectangular coordinate system. $L_{1}$ and $L_{2}$ are located at the same coordinate axis, and the distance between them is *b*. When the camera’s optical center is at position $L_{1}$, the projected position of point *P* on the imaging plane is $P_{1}$. When the camera’s optical center is at position $L_{2}$, the projected position of point *P* on the imaging plane is $P_{2}$. The other geometrical details are shown in Figure 3.

On the basis of Figure 3, the following proportional relationship exists in similar triangles:

(5) $$\frac{X_{1}}{X}=\frac{f}{Z},$$

(6) $$\frac{X_{2}}{X-b}=\frac{f}{Z}.$$

From Equations (5) and (6), we have

(7) $$Z=\frac{bf}{X_{1}-X_{2}}.$$

Thus, the target’s depth is obtained according to the information of the target in the image, the camera’s translation distance, and the camera’s focal length. When the depth is known, the other two coordinates of the target can be obtained as follows:

(8) $$X=\frac{bX_{1}}{X_{1}-X_{2}},$$

(9) $$Y=\frac{by_{1}}{X_{1}-X_{2}}.$$

 $X_{1}-X_{2}$ is called the parallax, and *b* is called the baseline.

The above method is commonly used for monocular vision 3D localization. Because the horizontal movement of the camera ensures constant coordinates along the y axis, the correction link is omitted, and then the image feature points are directly matched. By slightly moving the monocular camera, the principle of 3D target localization is similar to that of the binocular camera. However, there are also some limitations. First, the monocular camera must be mobile to obtain images from two places. Second, the results of target coordinates X,Y,Z are affected by parallax $X_{1}-X_{2}$. Therefore, calculation errors accumulate. The limitations of directly using a binocular camera are introduced in the last section.

### 2.3. Nonlinear Filter

The extended Kalman filter (EKF) is a classical nonlinear estimator that is widely applied for target tracking [43]. The EKF has three main steps, i.e., state prediction, Kalman gain calculation, and state correction. Its algorithmic structure is similar to that of the conventional linear KF. Since linearization in the EKF only uses the first-order term in the Taylor expansion, the EKF’s estimation performance for nonlinear target tracking is limited. Although the EKF has extended the KF method to nonlinear applications, EKF still has many disadvantages. In the EKF, the linearization error can hardly be avoided, and the divergence problem always happens. An improved nonlinear filter, the cubature Kalman filter (CKF) [44], is an algorithm that is approximately the closest to the Bayesian filter in theory. After strict mathematical derivation, the estimation accuracy and convergence of the CKF are guaranteed in theory. CKF refers to the idea of the particle filter. In the CKF, many particles with identical weights are selected for nonlinear function propagation processing according to the cubature criterion and the prior probability density distribution. Therefore, the calculation cost is low, there is no need to linearize the nonlinear function, and the linearization error is eliminated. The divergence problem in the EKF was also resolved.

## 3. Moving-Target Detection and Extraction

**Figure 4:**
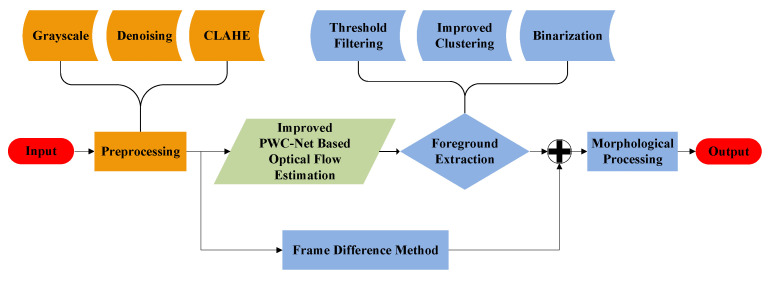
Moving-target detection and extraction process.

Moving-target tracking based on the monocular camera first needs to accurately capture a moving target in a video image. Therefore, this section proposes a moving-target detection method based on an improved PWC-Net, and a moving-target extraction method that is a combination of the improved K-means aggregation clustering algorithm, frame difference method, and morphological operation. The diagram of the proposed detection and extraction method is shown in Figure 4.

### 3.1. Video Image Preprocessing

#### 3.1.1. Grayscale Processing

Grayscale processing converts color images into grayscale images. Grayscale images can enhance and highlight image features. Because the expression of grayscale image information is simple, it can reflect the local and overall chromaticity with different digital values, brightness, and additional scene information. This paper uses the averaged weight method to complete the image processing. Different weights are allocated according to the three primary colors, i.e., red, green, and blue. Since people are sensitive to green and red, the weights of the grayscale processing are always set with special values [45,46]. In this paper, we define them as follows:

(10) g(i,j)=0.3R(i,j)+0.59G(i,j)+0.11B(i,j).

**Figure 5:**
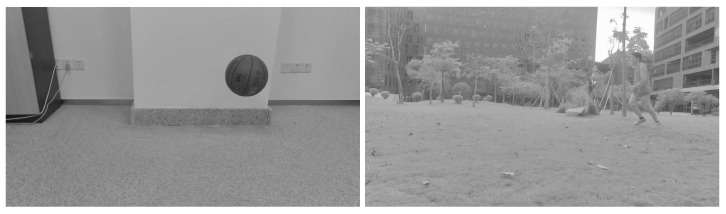
Images acquired after grayscale processing (an indoor basketball and an outdoor person).

Two groups of video images were collected. The two processed video images represent the different motions of different moving targets and motion states in different indoor and outdoor scenes. As shown in Figure 5, a basketball is falling and a target person is running, and the processed figures are applied for further target object extraction.

#### 3.1.2. Wavelet Transform Threshold for Noise Elimination

Noise exists in an image after grayscale processing, which impacts the final results since they have different characteristics after orthogonal wavelet decomposition. Thus, the wavelet transform threshold is applied to eliminate the noise. The purpose of this method is to find a suitable threshold that can guarantee appropriate wavelet coefficients. The applied wavelet transform threshold method for noise elimination has three steps. We define an actual signal as follows:

(11) q(t)=s(t)+e(t)

where s(t) is the effective signal, and e(t) is the noise.

The first step is to select the wavelet base. In terms of the wavelet base, we selected the Haar wavelet and then performed the orthogonal wavelet transform on the measured signal:

(12) $$WT_{q}\left(a,b\right)=WT_{s}\left(a,b\right)+WT_{e}\left(a,b\right).$$

The second step is to select the threshold and threshold processing function to determine the wavelet transform coefficients. We selected the soft threshold processing function:

(13) $$f_{\lambda }^{s}\left(w\right)=\left\{\begin{array}{c} ,|w|\geq \lambda \\ 0,|w|<\lambda \end{array}\right.$$

where *w* is the wavelet coefficient, and λ is the selected threshold. To set an appropriate threshold, we used

(14) $$\lambda =\sigma \sqrt{2\ln N}$$

*N* is the sum of the number of wavelet coefficients of the actual signal after wavelet transform decomposition, and σ is the standard deviation of the noise signal.

**Figure 6:**
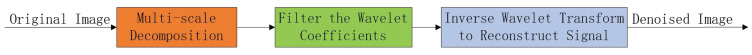
The process of the wavelet transform threshold for noise elimination.

**Figure 7:**
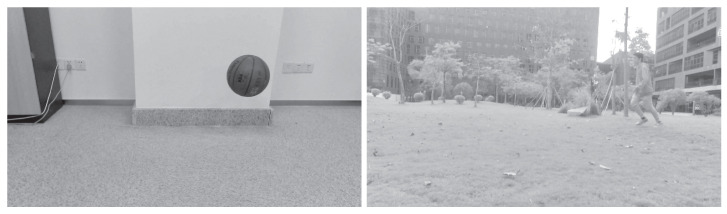
Images processed with the wavelet transform threshold method (original images are shown in Figure 5).

The third step is reconstructing the signal according to the low-frequency and high-frequency coefficients after wavelet decomposition. The process of the wavelet transform threshold method is shown in Figure 6. Then, the images in Figure 5 were processed using the wavelet transform threshold method, and the results are shown in Figure 7, indicating improved performance.

#### 3.1.3. Contrast Limited Adaptive Histogram

**Figure 8:**
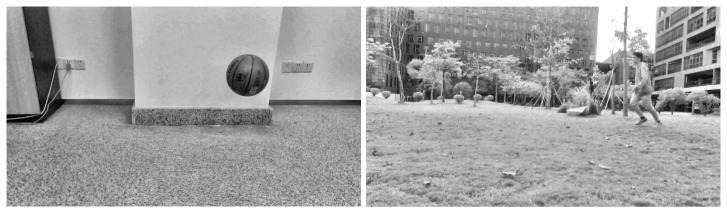
Final images after grayscale processing, noise elimination, and contrast enhancement.

Equalization (CLAHE) To further simplify the complexity for object extraction in the later optical flow step, we needed to enhance the image contrast. Thus, since the images had been processed during the previous two steps, i.e., grayscale processing and noise elimination, we directly utilized the CLAHE method [47] to improve the images. The CLAHE algorithm restricts the histogram of each sub-block region to an appropriate area to overcome the problem of overamplifying the noise. Referring to [48], the image processed by the CLAHE algorithm is shown in Figure 8.

#### 3.1.4. Optical Flow Estimation by Improved PWC-Net

**Figure 9:**
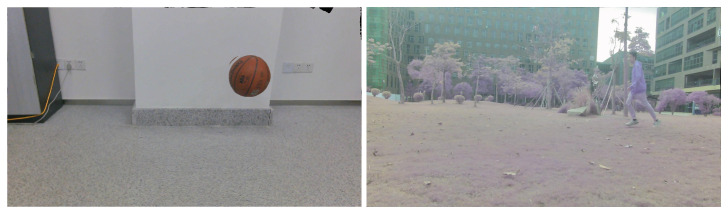
Problems of doubling images, ambiguity, and invalid information.

**Figure 10:**
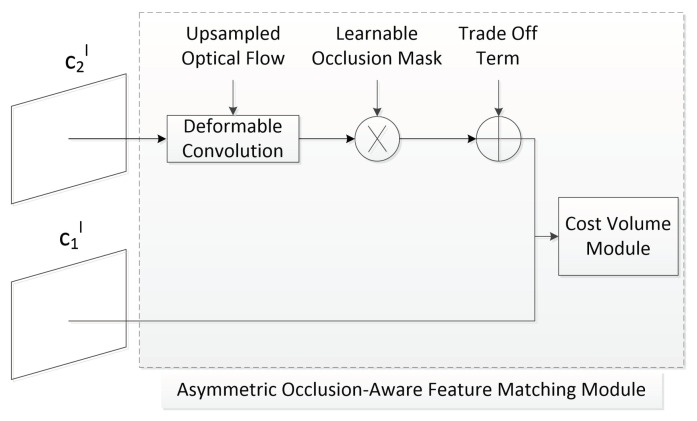
AsymOFMM’s overall structure in the improved PWC-Net.

With the pre-processed images, we next needed to identify a moving object by using the optical flow method before accurately extracting the target object. PWC-Net is a deep-learning optical flow estimation network proposed in 2018 [19]. However, in the PWC-Net method, the warping problem exists that results in doubling images, white space, ambiguity, and invalid information phenomena, as shown in Figure 9. To resolve the warping problem, we needed to detect the areas with the phenomena of doubling images, white space, ambiguity and invalid information, and eliminate them accurately. The asymmetric occlusion-aware feature matching module (AsymOFMM) that can learn occlusion masks was proposed in 2020 [21]. The AsymOFMM method can predict an occluded area and filter out useless information generated by warping without additional supervision with almost negligible calculation costs. AsymOFMM’s overall structure on the layer of the feature pyramid in the improved PWC-Net is shown in Figure 10.

The parameter training strategy of the improved PWC-Net was similar to that of PWC-Net. “Flying Chairs” was the basic training set, and the initial learning rate was equal to 0.0001. The batch size was 8 with 1.2 M iterations, and it halved the learning rate when the number of iterations reached 0.4, 0.6, 0.8, and 1 M. The FlyingThings3D dataset was finetuned. The initial learning rate was =0.00001 and batch size =4 for 0.5 M iterations. Then, the learning rate was halved when the number of iterations reached 0.2, 0.3, and 0.4 M. The loss function adopted the standard error measurement parameter in optical flow estimation, end-point error (EPE). The calculation formula of EPE is as follows:

(15) $$EPE=\Sigma \sqrt{{\left(u-u_{g}\right)}^{2}+{\left(v-v_{g}\right)}^{2}}.$$

where *u* and *v* are the components of each pixel’s predicted optical flow value in the transverse and longitudinal directions, and $u_{g}$ and $v_{g}$ are the components of the actual optical flow value in the label in the transverse and longitudinal directions.

As shown in Table 1, through many experiments on the Sintel, KITTI 2012, and KITTI 2015 datasets, the improved PWC-Net based on AsymOFMM substantially improved all datasets. In the table, AEPE refers to the average EPE of all effective pixels in the image, FL-all refers to the percentage of abnormal optical flow values in all effective pixels in the image, and the calculation formula of AEPE is as follows:

(16) $$AEPE=\frac{\Sigma \sqrt{{\left(u-u_{g}\right)}^{2}+{\left(v-v_{g}\right)}^{2})}}{mn},$$

where *m* and *n* are the numbers of pixels in the horizontal and vertical directions of the image, respectively.

**Figure 11:**
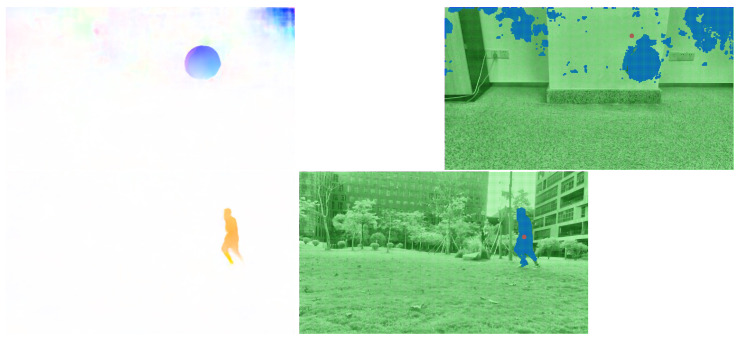
Results after PWC-Net.

We directly used PWC-Net to process the images in Figure 8 and acquire the results in Figure 11.

**Figure 12:**
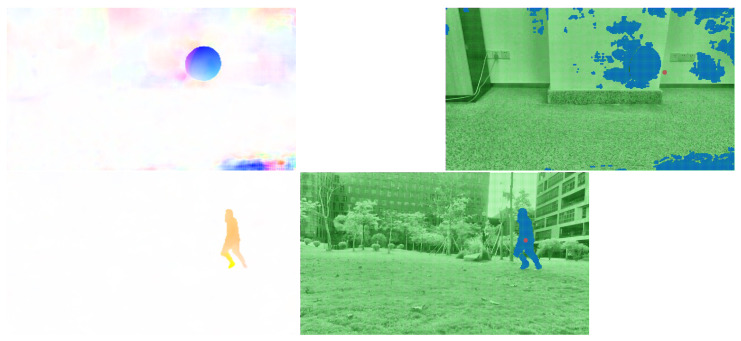
Calculation results of the improved PWC-Net.

The improved PWC-Net was used for optical flow prediction for two frame images. The two different visualization results are shown in Figure 12. Next, we needed to extract the moving objects for the processed optical flow images.

### 3.2. Target Extraction

**Figure 13:**
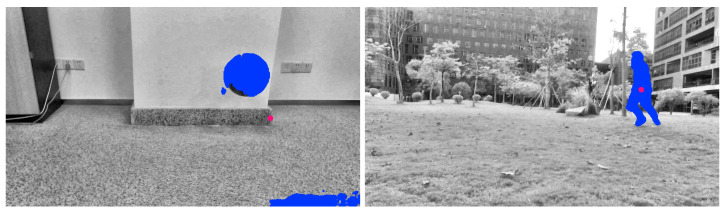
Threshold filtering results from Figure 12.

Next, we needed to accurately locate the position of the moving target in the 2D camera view from the processed optical flow images. However, in these images, the optical flow of the moving target was still mixed with some background edge values that interfered with the accuracy of extracting the moving target, as shown in the blue areas in Figure 12. This paper proposes a target extraction strategy using an improved K-means and aggregation clustering algorithm combined with a frame difference method and morphological operation to eliminate useless background optical flow. In addition, to improve the efficiency, we first set a threshold for the optical flow values to filter interesting dynamic information from the optical flow image in Figure 12. The optical flow after simple threshold filtering is shown in Figure 13 as an example. Then, the proposed 2-step extraction method was applied.

#### 3.2.1. Improved K-Means and Agglomerative Clustering Algorithm

On the basis of the improvement of the K-means and agglomerative clustering algorithm, the proposed algorithm was divided into two stages to cluster the optical flow samples combined with the advantage of not specifying the number of categories before clustering.

At the first stage, the key difference between the classical K-means algorithm and the proposed improved method is in the initialization process of K cluster centers. Specific conditions were added to the selection process of cluster centers to lengthen the distance between cluster centers enough to avoid the adverse effects caused by completely random initialization. First, at the first stage of the improved clustering algorithm, the corresponding sample features are established for each filtered optical flow in the optical flow field for similarity measurements. Specifically, suppose that, in the pixel coordinate, the position coordinates of the *j*-th optical flow vector in the image ${\vec{X}}_{j}$ are $\left(x_{j},y_{j}\right)$, the corresponding optical flow amplitude is $A_{j}$, and the optical flow direction is $D_{j}$. Then, we define four-dimensional sample features $X_{j}$ of the *j*-th optical flow vector as follows:

(17) $${\vec{X}}_{j}=\left[A_{j}\;D_{j}\;X_{j}\;y_{j}\right].$$

To simplify the calculation, normalization processing is required. The normalization has

(18) $$\left\{\begin{array}{c} \tilde{A_{J}}=A_{j}/A_{max}\\ \tilde{D_{J}}=D_{j}/D_{max}\\ \tilde{X_{J}}=X_{j}/\sqrt{x_{max}^{2}+y_{max}^{2}}\\ \tilde{y_{J}}=y_{j}/\sqrt{x_{max}^{2}+y_{max}^{2}}. \end{array}\right.$$

Similarly, the construction of other optical flow vector sample features was completed. Second, Euclidean distance was selected as the similarity measurement index at the first stage of the proposed improved clustering method. Specifically, once again, we defined one of the categories in the clustering process as $S_{k}$ with cluster center sample features $C_{k}$. Then, the similarity between the *j*-th optical flow vector in image ${\vec{X}}_{j}$ and the cluster category was $S_{k}$, expressed as the Euclidean distance between sample features $X_{j}$ and the cluster center sample features $C_{k}$:

(19) $$d_{jk}=\left|\right|X_{j}-C_{k}\left|\right|=\sqrt{\Sigma_{i=1}^{4}{\left(X_{ji}-C_{ki}\right)}^{2}}.$$

A small distance denotes a high similarity between the optical flow vector and a class.

Lastly, the numbers of clusters and their cluster centers were automatically determined by using the sample features and similarity measurement function defined above, and the optical flow clustering in the first stage was completed by combining it with the classical K-means algorithm. The specific steps are as follows:

(1) Initialize the first cluster center. Set the first optical flow class $S_{1}$, randomly select a filtered optical flow vector (blue parts in Figure 13) assigned to $S_{1}$ and as the center of the $S_{1}$, and assume its sample features as the central sample features of category $C_{1}$.
(2) Calculate the similarity. Select an unclassified optical flow $\vec{X}$ in the optical flow field, calculate its similarity with the current existing clustering class, and record the Euclidean distance between its sample features and the sample features of the center of its most similar category.
(3) Optical flow classification. Set a threshold of *T*. If d>T, we set a new clustering class and classify the corresponding optical flow $\vec{X}$ into the new class to serve as the center of the new class. If d≤T, we classify the corresponding optical flow into its most similar class, calculate the average value of all the optical flow sample features in this class, and take the average value as the central sample features of this class to complete the update of the center.
(4) For each other unclassified optical flow in the optical flow field, repeat Steps 2 and 3 until the cluster center does not change, and output the final K cluster centers and corresponding cluster members.

**Figure 14:**
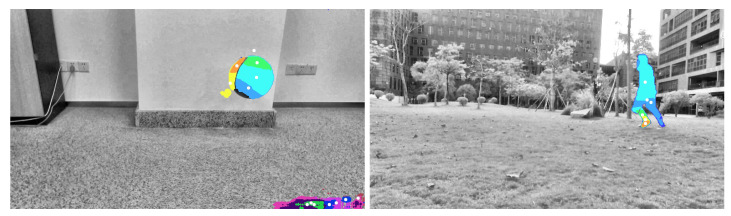
Calculation results of the first stage of the clustering algorithm.

The optical flow eliminated by the first stage of the clustering algorithm in this paper is shown in the figure below. Thus, the sample data in blue in Figure 13 were divided into the differently colored clusters in Figure 14. The white dot in the figure is the clustering center of each class. The foreground optical flow was aggregated in a large area due to the similar characteristics, while the background optical flow was scattered into many categories due to their differences.

At the second stage of the improved algorithm, the idea of the cohesion method in hierarchical clustering is used. After a certain number of clusters are obtained in the first stage, some clusters should be merged, and the number of clusters can lastly be optimized. We propose a modified method to optimize the cluster number that reproduces some data and refers to the cohesion method. Unlike the classical cohesion method, the proposed method reproduced some assist points to improve clustering performance.

(1) Eight new data samples were calculated according to the position of each cluster center. The eight new data points were centered on one cluster center and arranged at a certain angle. Assuming that the cluster central position is $\left(C_{xk},C_{yk}\right)$, the new produced data point is $\left(m_{i},n_{i}\right)$, which has
  (20) $$\begin{array}{c} m_{i}=C_{xk}+\cos\left(angle\left[i\right]\right)\times number\\ n_{i}=C_{yk}-\sin\left(angle\left[i\right]\right)\times number \end{array}$$
  where, $angle=[0^{\circ },45^{\circ },90^{\circ },135^{\circ },180^{\circ },225^{\circ },270^{\circ },315^{\circ }]$;
(2) Then, the distances between all newly generated data points from two different clusters (for example, 8 points for Cluster 1 and another 8 points from Cluster 2) are calculated, and the shortest distance is selected as the inter-class distance.
(3) Set the threshold and merge the two categories whose inter-class distance between two clusters is less than the threshold.
(4) Update and return the final cluster center and the cluster member.

**Figure 15:**
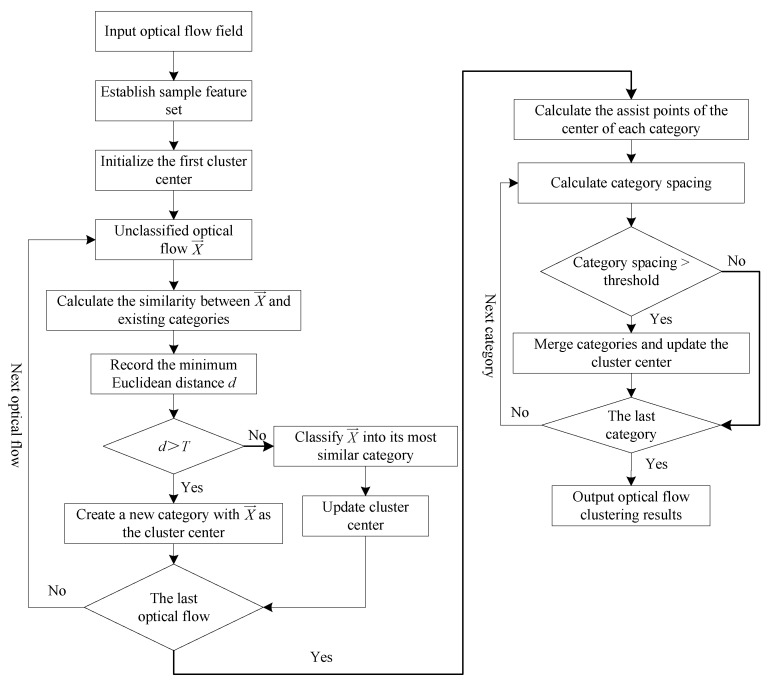
Calculation flow of the improved clustering algorithm.

The overall flow of the optical flow clustering algorithm in this paper is shown in Figure 15.

**Figure 16:**
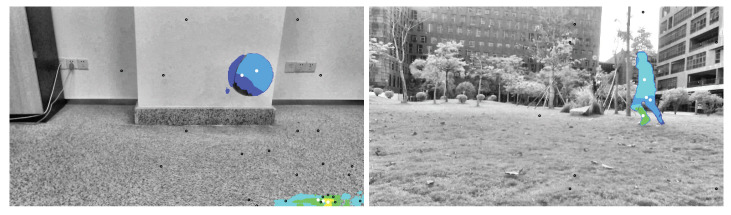
Calculation results of the second stage of the clustering algorithm.

**Figure 17:**
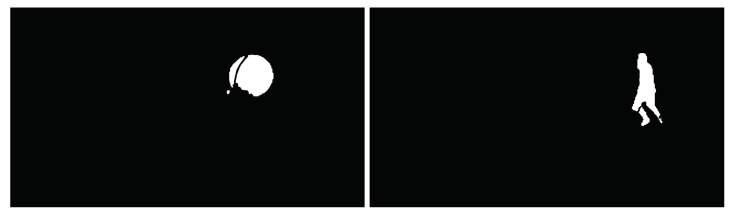
Final calculation result of the improved clustering algorithm.

The optical flow calculated by the improved clustering algorithm is shown in Figure 16. Few clusters existed, and they could help in target extraction. Next, after setting a threshold, most optical flow noise can be effectively eliminated through a binarization step. The final optical flow is shown in Figure 17; thus, only true moving-target information remained.

#### 3.2.2. Accurate Extraction Based on Frame Difference Method and Morphological Operation

Figure 17 results show that the target areas still had some black parts that divided the true targets into multiple parts. We extracted the final moving target with an extra step to improve the accuracy.

**Figure 18:**
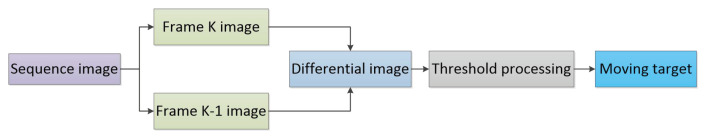
Work flowchart of two frame difference method.

The frame difference method is commonly used for moving-target detection and segmentation. The flow chart of the two-frame difference method is shown in Figure 18. The *n*-th and n−1-th frames in a video are recorded as $\gamma_{n}$ and $\gamma_{n-1}$, respectively, and the gray values of the corresponding pixel points of the two frame images are recorded as $\gamma_{n}\left(x,y\right)$ and $\gamma_{n-1}\left(x,y\right)$, respectively. Here, (x,y) represents the positions of the pixel points in the image. When calculating a difference image, the gray values of corresponding pixel points at each position on the two frame images are successive. Then, we can obtain the absolute value of image difference $D_{n}\left(x,y\right)$:

(21) $$D_{n}\left(x,y\right)=\left|\gamma_{n}\left(x,y\right)-\gamma_{n-1}\left(x,y\right)\right|.$$

Next, we set another threshold value for the gray value difference, binarized each pixel’s gray value difference according to Equation (22), and lastly obtained binarized image $R_{n}^{{}^{\prime }}\left(x,y\right)$.

(22) $$R_{n}^{{}^{\prime }}\left(x,y\right)=\left\{\begin{array}{c} 255,\;D_{n}\left(x,y\right)>\epsilon \\ 0,\;otherwise. \end{array}\right.$$

**Figure 19:**
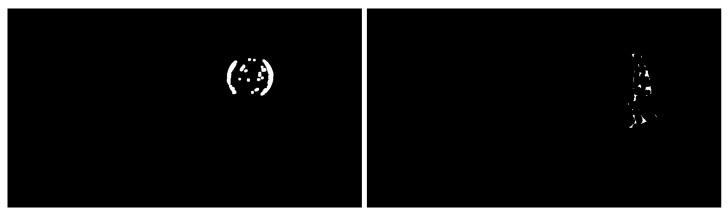
The value difference image of the inter-frame difference method.

Thus, the difference between two successive images is shown in Figure 19.

**Figure 20:**
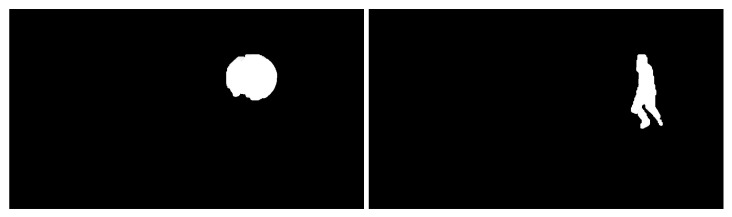
Fusion results of morphological processing and the frame difference method.

**Figure 21:**
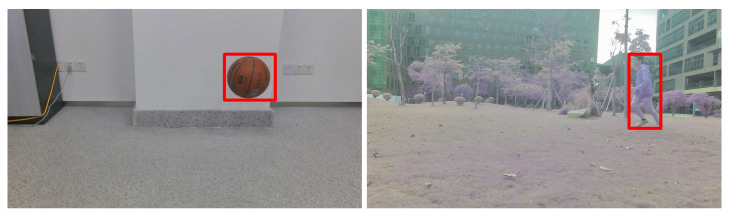
Moving target extraction results.

Then, morphological processing was applied [49] that eliminated the thorns, and filled the narrow discontinuities and small holes in the image using the results in Figure 17 and Figure 19. In other words, the target areas in Figure 17 and Figure 19 were added together. The region of the interested target after morphological processing and frame difference fusion is shown in Figure 20. The extraction strategy proposed in this paper could separate the background and moving-target optical flow well. Figure 21 shows the final extracted moving target.

## 4. Target Localization Based on Pinhole Imaging Theory

After the moving target is detected and extracted, the location of the moving target can be calculated. This paper proposes a simple mathematical model based on pinhole imaging theory. The target position relative to the camera can be calculated only by using the camera parameters and the measured two-dimensional image. Camera installation information was required, such as the camera’s position, and the camera lens was horizontal.

### 4.1. Localization Problem Formulation

**Figure 22:**
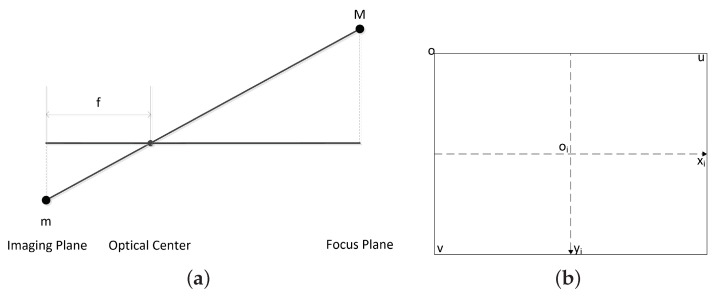
(**a**) Principle of camera imaging; (**b**) image coordinate system and pixel coordinate system.

The pinhole imaging model is the camera imaging principle shown in Figure 22a. An actual object point *M* in the 3D space had a corresponding image point m on the imaging plane after passing through camera optical center *C*. A specific proportional relationship exists between the size of the true object and its image. According to the Gaussian imaging formula, we have

(23) $$\frac{1}{f}=\frac{1}{a}+\frac{1}{b}$$

where *f* is the camera’s focal length, *a* is the object distance, and *b* is the image distance of the object. Since the object distance is much larger than the image distance, Equation (23) can be approximated as follows:

(24) $$\frac{1}{f}=\frac{1}{b}.$$

Assuming that the imaging plane coincided with the focal plane was reasonable, since the camera was horizontally installed. The distance between the imaging plane and the optical center was equal to the focal length f of the camera.

Four coordinates are usually mentioned in the proposed localization algorithm, i.e., the camera coordinate, world coordinates, image coordinate, and pixel coordinate. The camera coordinate uses $\left(X_{c},Y_{c},Z_{c}\right)$ to represent a point position, and the origin is the camera. The world coordinate is denoted by (X,Y,Z). $\left(X_{i},y_{i}\right)$ presents a position in the image coordinate. In the pixel coordinate, the position is represented by (u,v).

The difference between the image and pixel coordinate systems is shown in Figure 22b. The origin of the image coordinate is $o_{i}$, and in the pixel coordinate, the origin is denoted by $\left(u_{0},v_{0}\right)$. These two coordinates have the same system, but with different scalars. Under the assumption that the side length of each pixel in the image is *d*, the following relationship between the image and the pixel coordinates obtained from Figure 22b takes the following form:

(25) $$X_{i}=\left(u-u_{0}\right)d,$$

(26) $$y_{i}=\left(v-v_{0}\right)d.$$

Expression (25) and Equation (26) can be rewritten as follows:

(27) $$\left[\begin{array}{c} u\\ v\\ x \end{array}\right]=\left[\begin{array}{ccc} \frac{1}{d} & 0 & u_{0}\\ 0 & \frac{1}{d} & v_{0}\\ 0 & 0 & 1 \end{array}\right]\left[\begin{array}{c} X_{i}\\ y_{i}\\ 1 \end{array}\right]$$

**Figure 23:**
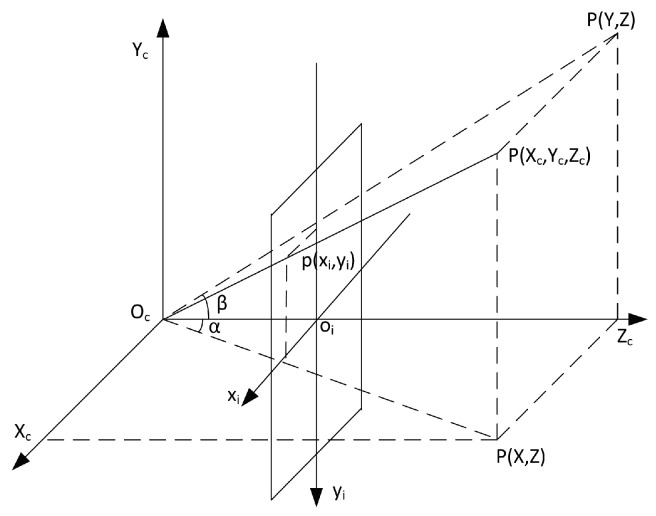
Visual coordinate system.

In order to simplify the calculation and analysis, we built a pinhole imaging model as shown in Figure 23. A new imaging plane at focal length f from the optical center of the camera was built; thus, 3D points were projected onto the camera coordinate system. Specifically, as shown in Figure 23, $O_{c}X_{c}Y_{c}Z_{c}$ is the camera coordinate system, $o_{i}X_{i}y_{i}$ is the image coordinate system, $O_{c}$ is the optical center, and the $Z_{c}$ axis coincided with the camera’s optical axis. $P(X_{c},Y_{c},Z_{c})$ is the point position in the 3D space of the camera coordinate system, and $p(X_{i},y_{i})$ is the 3D point’s projected position in the image coordinate system.

According to Figure 23, combined with Equation (24), $P(X_{c},Y_{c},Z_{c})$ and $p(X_{i},y_{i})$ have the following relationship:

(28) $$\frac{X_{c}}{X_{i}}=\frac{Z_{c}}{f},$$

(29) $$\frac{Y_{c}}{Y_{i}}=\frac{Z_{c}}{f}.$$

We have

(30) $$Z_{c}\left[\begin{array}{c} X_{i}\\ y_{i}\\ 1 \end{array}\right]=\left[\begin{array}{cccc} f & 0 & 0 & 0\\ 0 & f & 0 & 0\\ 0 & 0 & 1 & 0 \end{array}\right]\left[\begin{array}{c} X_{c}\\ Y_{c}\\ Z_{c}\\ 1 \end{array}\right]$$

On the basis of Equations (27) and (30), we obtain

(31) $$Z_{c}\left[\begin{array}{c} X_{i}\\ y_{i}\\ 1 \end{array}\right]=\left[\begin{array}{ccc} \frac{1}{d} & 0 & u_{0}\\ 0 & \frac{1}{d} & v_{0}\\ 0 & 0 & 1 \end{array}\right]\left[\begin{array}{cccc} f & 0 & 0 & 0\\ 0 & f & 0 & 0\\ 0 & 0 & 1 & 0 \end{array}\right]\left[\begin{array}{c} X_{c}\\ Y_{c}\\ Z_{c}\\ 1 \end{array}\right].$$

Let $M=\left[\begin{array}{ccc} \frac{1}{d} & 0 & u_{0}\\ 0 & \frac{1}{d} & v_{0}\\ 0 & 0 & 1 \end{array}\right]\left[\begin{array}{cccc} f & 0 & 0 & 0\\ 0 & f & 0 & 0\\ 0 & 0 & 1 & 0 \end{array}\right]$. Then, Equation (31) is simplified into

(32) $$Z_{c}=\left[\begin{array}{c} u\\ v\\ 1 \end{array}\right]=M\left[\begin{array}{c} X_{c}\\ Y_{c}\\ Z_{c}\\ 1 \end{array}\right].$$

Equation (32) shows that, for a coordinate point in 3D space, a unique pixel point can be found in its imaging plane. However, depth information cannot be obtained. Therefore, additional information is required, since we used monocular vision for 3D localization. Therefore, this paper proposes a strategy using the camera’s installation height to calculate the final 3D position that includes two steps: (1) computing the target azimuth angle and depth measurements, and (2) calculating the target Cartesian position using the measurements.

### 4.2. Angle and Distance Measurements

In the camera coordinate, azimuth and pitch/elevation angles are defined as α and β, respectively, and are shown in Figure 23 and have

(33) $$\alpha =\arctan\frac{X_{c}}{Z_{c}},$$

(34) $$\beta =\arctan\frac{Y_{c}}{Z_{c}}.$$

Substituting (28) and (29) into (33) and (34) yields

(35) $$\alpha =\arctan\frac{X_{i}}{f}=\arctan\frac{(u-u_{0})d}{f},$$

(36) $$\beta =\arctan\frac{y_{i}}{f}=\arctan\frac{(v-v_{0})d}{f}.$$

**Figure 24:**
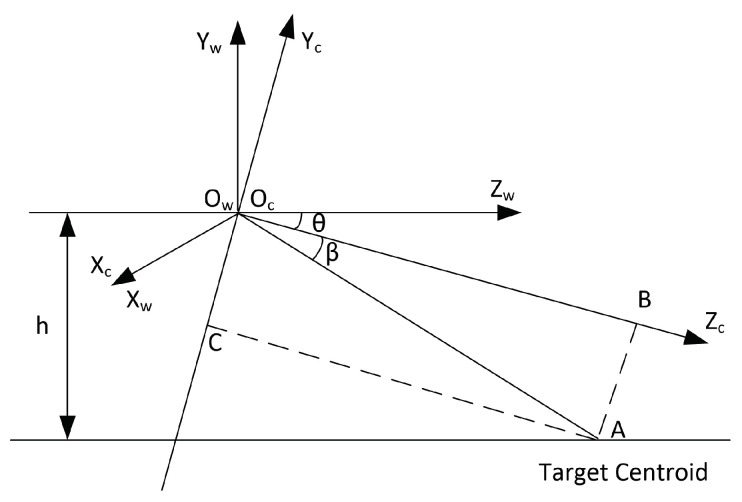
Projection diagram of distance information measurement.

Therefore, according to the position of the object point in the image plane, the angular information of the target object relative to the camera in 3D space can be roughly obtained. Angular information is acquired by only using the camera pixel image, pre-known visual size, and focal length. Lens distortion was ignored, or the noise caused by lens distortion satisfied a Gaussian distribution. However, it is still impossible to calculate the target depth by only using the azimuth and pitch angles. Additional reference information is required, e.g., the camera installation height. As shown in Figure 24, the camera was installed at height h from the ground. Moreover, the camera was placed manually, and its observation direction was known, defined as θ. The world coordinate system was established simultaneously, and the origin of the two coordinate systems coincided. For example the angular diagram for when the target moved on the ground is shown in Figure 24.

The relationship between the world coordinate system and the camera coordinate systems can be expressed in the following form:

(37) $$\left[\begin{array}{c} X_{c}\\ Y_{c}\\ Z_{c} \end{array}\right]=\left[\begin{array}{ccc} 1 & 0 & 0\\ 0 & \cos\theta & -\sin\theta \\ 0 & \sin\theta & \cos\theta \end{array}\right]\left[\begin{array}{c} X_{w}\\ Y_{w}\\ Z_{w} \end{array}\right]$$

From Equations (28) and (29), we can obtain the 2D image position of the target centroid, $\left(X_{i},y_{i}\right)$. Since the camera focal length *f* and the target centroid’s Y-axis position in the camera coordinate system are both known, the target centroid position $\left(X_{c},Y_{c},Z_{c}\right)$ can be calculated. Then, the distance between the target and the camera can be obtained,

(38) $$S=\sqrt{X_{c}^{2}+Y_{c}^{2}+Z_{c}^{2}}.$$

Since camera tilt angle θ is known, and the target centroid pitch angle can be obtained from Equation (36), according to the geometric relationship, Figure 24 shows

(39) $$O_{c}A=\frac{h}{\sin\theta +\beta },$$

(40) $$AB=O_{c}A\sin\left|\beta \right|.$$

In particular, when β=0, that is, $y_{i}=0$ is satisfied, $Y_{c}=0$ and $Z_{c}=\frac{h}{\sin\theta }$ can be easily obtained from Figure 24. At that time, $X_{c}=\frac{Z_{c}X_{i}}{f}$ is acquired from Equation (30). In addition, when the height of the target cannot be ignored (i.e., the target is not mass on the ground), $O_{c}A=\frac{h-l}{\sin(\theta +\beta )}$ exists. Accordingly, when β=0 holds, $Z_{c}=\frac{h-l}{\sin\theta }$ can be known, where *l* is the height of the target centroid from the ground. The target height can be estimated using some known or pre-given reference static object.

## 5. Moving-Target Tracking Using Visual Image and Cubature Kalman Filter

The discrete state transition and observation equations of the nonlinear system in this paper take the following form:

(41) $$\begin{array}{cc} \; & X_{k}=f\left(X_{k-1}\right)+\omega_{k}\\ \; & Z_{k}=h\left(X_{k}\right)+\zeta_{k}, \end{array}$$

where $X_{k}$ is the state vector at time instant *k*, $Z_{K}$ is the measurement vector at *k*, f(·) is the dynamic model function, h(·) is the measurement function. Here, we used both angle and distance measurements. $\omega_{k},\zeta_{k}$ were assumed to be Gaussian white noise with zero additive mean, which represents the process and measurement noise of which the covariance matrices are $Q_{k},R_{k}$, respectively.

Since the CKF has many advantages in nonlinear estimation [44], it has been widely applied as a state-of-the-art method. The structure of the CKF algorithm is divided into two parts, i.e., prediction and update with measurement steps.

**Prediction update**:

(i) Decompose estimation error covariance matrix.
  (42) $$P_{k|k}=S_{k|k}{\left(S_{k|k}\right)}_{T}.$$
(ii) Calculate the cubature point.
  (43) $$X_{i,k|k}=S_{k|k}{\mathrm{xi}}_{i}+{\hat{X}}_{k|k},\;i=1,2,\Lambda \;m.$$
(iii) Calculate the propagation cubature point of the state transfer function.
  (44) $$X_{i,k+1|k}^{*}=f_{K}\left(X_{i,k|k}\right),\;i=1,2,\cdots ,m,$$
  where $X_{i,k|k}$ and $X_{i,k+1|k}^{*}$ are cubature points, *m* is the number of cubature points. When using the third-order spherical radial criterion, the number of cubature points should be twice the dimension n of the state vector of the nonlinear system. $\xi_{i}$ is the cubature point set, $\xi_{i}=\sqrt{\frac{m}{2}}\left[1\right]$. i=1,2,...,m. [1] is the point set of *n*-dimensional space, where $\left[1\right]=\left[\begin{array}{c} \left(\begin{array}{c} 1\\ 0\\ \vdots \\ 0 \end{array}\right),\left(\begin{array}{c} 0\\ 1\\ \vdots \\ 0 \end{array}\right),\cdots ,\left(\begin{array}{c} 0\\ 0\\ \vdots \\ 1 \end{array}\right),\left(\begin{array}{c} -1\\ 0\\ \vdots \\ 0 \end{array}\right),\left(\begin{array}{c} 0\\ -1\\ \vdots \\ 0 \end{array}\right),\cdots ,\left(\begin{array}{c} 0\\ 0\\ \vdots \\ -1 \end{array}\right) \end{array}\right]$.
(iv) Calculate status prediction value.
  (45) $${\hat{X}}_{k+1|k}=\frac{1}{m}\sum_{i=1}^{m}X_{i,k+1|k}^{*}.$$
(v) Calculate the prediction covariance matrix.
  (46) $$P_{k+1|k}=\frac{1}{2n}\sum_{i=1}^{2n}X_{i,k+1|k}^{*}{\left(X_{i,k+1|k}^{*}\right)}^{T}-{\hat{X}}_{k+1|k}{\left(\hat{k+1|k}\right)}^{T}+Q_{k}.$$

Update with measurement:

(i) Decompose the prediction covariance matrix.
  (47) $$P_{k+1|k}=S_{k+1|k}{\left(S_{k+1|k}\right)}^{T}.$$
(ii) Calculate the updated cubature point.
  (48) $$X_{i,k+1|k}=S_{k+1|k}\xi_{i}+{\hat{X}}_{k+1|k},\;i=1,2,\cdots ,m.$$
(iii) Calculate the propagation cubature point of the measurement function.
  (49) $$Z_{i,k+1|k}=h_{K+1}\left(X_{i,k+1|k}\right),\;i=1,2,\cdots ,m.$$
(iv) Calculate the measured predicted value.
  (50) $${\hat{Z}}_{k+1|k}=\frac{1}{m}\sum_{i=1}^{m}Z_{i,k+1|k}.$$
(v) Calculate the innovation,
  (51) $$e_{K+1}=Z_{K+1}-{\hat{Z}}_{k+1|k}$$
  where $Z_{k+1}$ is the measured value at k+1.
(vi) Calculate innovation covariance matrix.
  (52) $$P_{ZZ,k+1|k}=\frac{1}{2n}\sum_{i=1}^{2n}Z_{i,k+1|k}{\left(Z_{i,k+1|k}\right)}^{T}-{\hat{Z}}_{k+1|k}{\left({\hat{Z}}_{k+1|k}\right)}^{T}+R_{k}.$$
(vii) Calculate the cross covariance matrix.
  (53) $$P_{XZ,k+1|k}=\frac{1}{2n}\sum_{i=1}^{2n}X_{i,k+1|k}{\left(Z_{i,k+1|k}\right)}^{T}-{\hat{X}}_{k+1|k}{\left({\hat{Z}}_{k+1|k}\right)}^{T}.$$
(viii) Calculate the cubature Kalman filter gain.
  (54) $$K_{k+1}=P_{XZ,k+1|k}{\left(P_{ZZ,k+1|k}\right)}^{-1}$$
(ix) Calculate the estimated state value at k+1.
  (55) $${\hat{X}}_{k+1|k+1}={\hat{X}}_{k+1|k}+K_{k+1}\left(Z_{K+1}-{\hat{Z}}_{k+1|k}\right).$$
(x) Calculation of estimation error covariance matrix.
  (56) $$P_{k+1|k+1}=P_{k+1|k}-K_{k+1}P_{ZZ,k+1|k}K_{k+1}^{T}.$$
  On the basis of the pinhole imaging and positioning model described in Section 4, the CKF is used to estimate the 3D target state compared with the assumed dynamic models.

## 6. Experimental Examples

In this paper, we used an ordinary monocular industrial camera to acquire some videos, and the detailed parameters of the camera are shown in Table 2. The methods proposed in this paper were verified step by step. More specifically, the improved optical flow method introduced in Section 3 is first verified, and then the proposed geometrical algorithm using the pinhole imaging method is demonstrated. Lastly, combined with the CKF algorithm, we verify the effectiveness of the proposed strategy in Section 3, Section 4 and Section 5 via different examples with some comparisons.

### 6.1. Target Detection and Extraction (Using the Proposed Method in Section 3)

**Figure 25:**
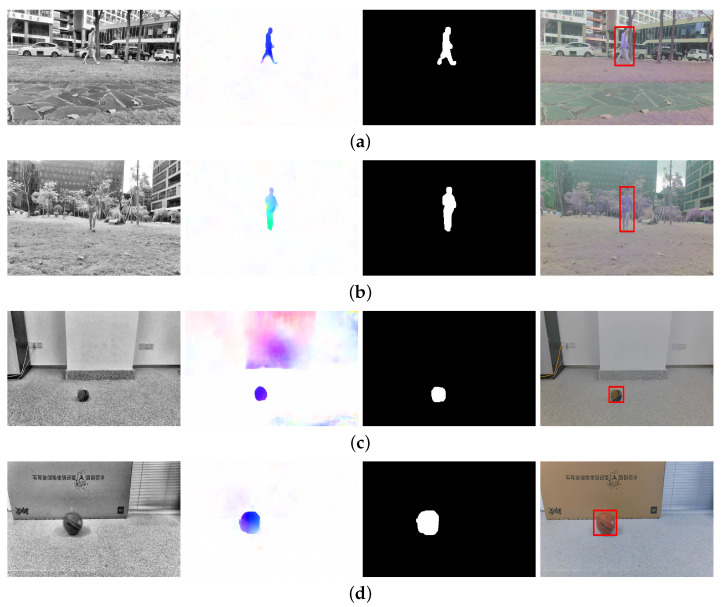

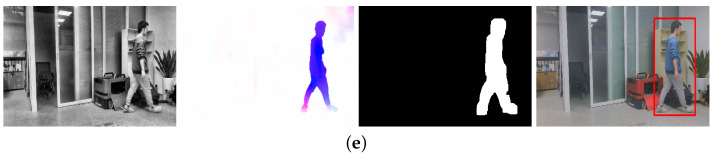
Experimental results of moving target detection and extraction. (**a**) A walking person outside; (**b**) a running person closer; (**c**) a small static ball indoors; (**d**) moving basketball indoors; (**e**) a walking person indoors.

Several moving target videos with different target objects and motion states were randomly taken in indoor and outdoor environments. The optical flow calculation and moving-target extraction network using the proposed algorithm could effectively suppress the interference of background noise and accurately extract the region of interest. The experimental results are shown in Figure 25. The first column shows the pre-processed images, the second column indicates the optical flow visualization images, the third column shows the moving target segmentation results, and the fourth column displays the final extracted moving targets.

### 6.2. Moving Target Localization (Using the Proposed Methods in Section 4 and Section 5)

In order to verify the accuracy of the positioning method, in this experiment, several identified target images were captured. Then, the data were calculated, and we lastly compared the results with the true values measured with an advanced RGB camera. The first step was to set up some experimental scenarios and randomly place the experimental target in multiple positions. The camera’s height, shooting angle, and target position were different. In the second step, we recorded the relevant data needed for model calculation, such as the camera’s height and shooting angle, collected the target image, and extracted target information from the 2D image by using the proposed algorithm in Section 3; then, we calculated the targets’ locations. Six pictures captured from the previous videos in Figure 25 were used. In the last step, the angle and distance measurements were used to calculate the 3D target positions of different examples. The experimental results are shown in Table 3 [42]. Table 3 shows that the target’s locations computed with the proposed geometrical method were very close to the true values, but some errors still existed that had been caused by the extraction process and camera lens distortion noise, which commonly satisfy Gaussian distribution.

The modified CKF method was applied to process the measurements to improve the target tracking performance. The CKF utilized our contribution regarding the modified measurement model proposed in (39) and (40). In the CKF algorithm, we assumed that the target moved with constant velocity, and in each image, multiple angular measurements could be produced to guarantee the CKF running. In the CKF simulation, the sampling time interval was *T*. With the proposed pinhole imaging localization method, the state vector contained the 3D position and velocity of the moving target. The measurement was the 2D target position in the image coordinate system. Therefore, the dynamic system model has

(57) $$X_{k}={\left[\begin{array}{c} X_{c}\\ Y_{c}\\ Z_{c}\\ V_{X}\\ V_{Y}\\ V_{Z} \end{array}\right]}_{k}=\left[\begin{array}{cccccc} 1 & 0 & 0 & T & 0 & 0\\ 0 & 1 & 0 & 0 & T & 0\\ 0 & 0 & 1 & 0 & 0 & T\\ 0 & 0 & 0 & 1 & 0 & 0\\ 0 & 0 & 0 & 0 & 1 & 0\\ 0 & 0 & 0 & 0 & 0 & 1 \end{array}\right].{\left[\begin{array}{c} X_{c}\\ Y_{c}\\ Z_{c}\\ V_{X}\\ V_{Y}\\ V_{Z} \end{array}\right]}_{k-1}+G\omega_{k-1},$$

and the measurement model is

(58) $$Z_{k}={\left[\begin{array}{c} u\\ v \end{array}\right]}_{K}=h\left({\left[X_{k}\right]}_{k}\right)+\zeta_{k}=\left[\begin{array}{c} \frac{fX_{C}}{dZ_{C}}+u_{0}\\ \frac{fY_{C}}{dZ_{C}}+v_{0} \end{array}\right]+\zeta_{k}$$

 $\omega_{k-1}$ and $\zeta_{k}$ are the process and measurement noise, respectively. G is a transformation matrix.

To clearly show the advantages of the proposed CKF, a moving target with nonlinear process noise (maneuvering) was tracked. In the dynamic model, we have

$G={\left[\begin{array}{cccccc} 0 & 0 & 0 & 0 & 0 & 0\\ 0 & \sin[\vartheta \frac{k-1}{T}] & 0 & 0 & 0 & 0\\ & & & 0 & \end{array}\right]}_{6\times 6}$, $\omega_{k-1}∼N\left(0,Q_{K-1}\right)$, and $Q_{k-1}={\left[\begin{array}{cccccc} 0 & 0 & 0 & 0 & 0 & 0\\ 0 & \tau & 0 & 0 & 0 & 0\\ & & & 0 \end{array}\right]}_{6\times 6}.$ ϑ and τ determine the amplitude and frequency, respectively, of the moving target during sinusoidal motion. The EKF method [50,51] was applied as a comparison, and both the EKF and CKF were applied with 1000 Monte Carlo repeats.

In this part, Examples 1 and 2 are provided with different target initial positions (EKF and CKF had the same initial state). In Example 1, the true target initial state was ${\left[300,400,-800,2,4,6\right]}^{T}$. The initial states are $\left[X_{0}={\left[320,420,-820,2.1,3.8,6.3\right]}^{T}\right]$, $R_{k}=\mathrm{diag}\left[1,1\right]$ and $P_{0}=\mathrm{diag}\left[100,100,100,100,100,100\right]$ for both Kalman filters. The sampling time was reset to 0.15 s, and the total running time was 300 s. To ensure the sinusoidal motion of the moving target, we set amplitude ϑ=600 m in the noise driving matrix, and the frequency τ=0.12 s in the process noise variance.

**Figure 26:**
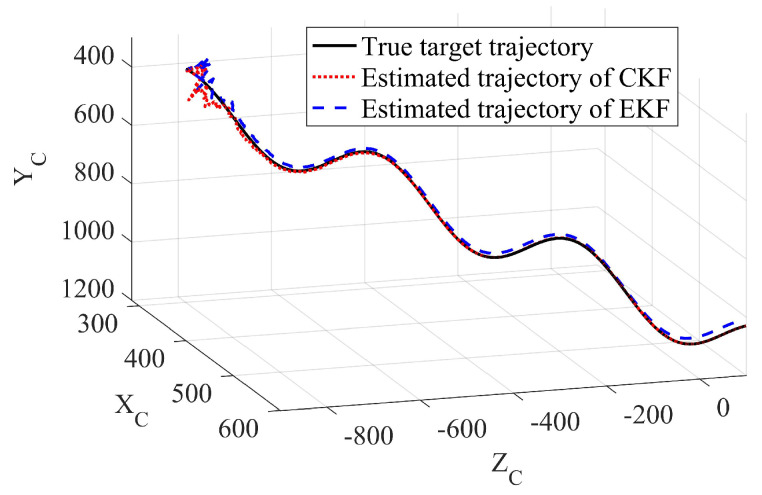
Comparison of EKF and CKF for tracking nonlinear mobile target, unit: meters (Example 1).

**Figure 27:**
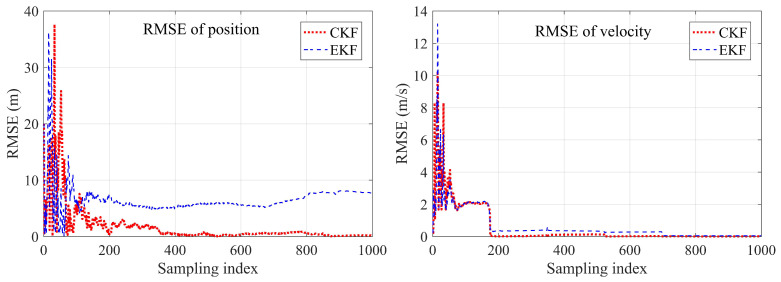
Comparison of the position and velocity RMSEs between EKF and CKF of Example 1.

**Figure 28:**
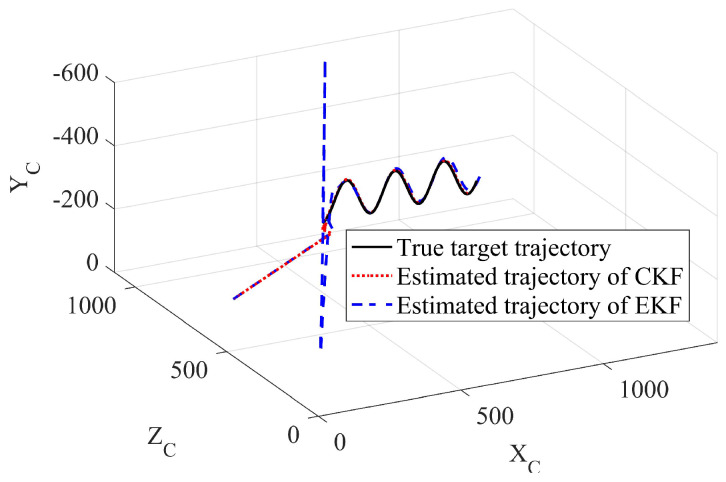
Comparison of EKF and CKF for tracking nonlinear mobile target, unit: meters (Example 2).

**Figure 29:**
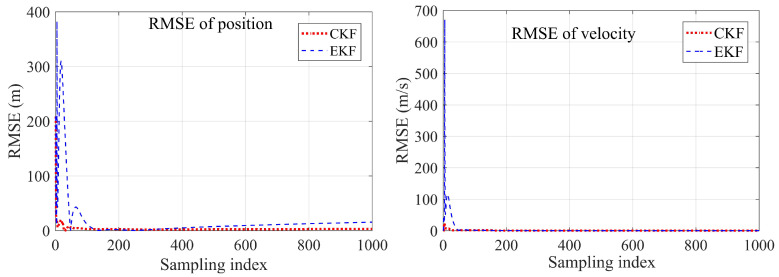
Comparison of the position and velocity RMSEs between EKF and CKF of Example 2.

In Example 2, the true target initial state was ${\left[-800,300,400,1,1,2\right]}^{T}$. The initial states is ${\left[100,-100,630,4.2,0.8,2.2\right]}^{T}$, $R_{k}=\mathrm{diag}\left[1,1\right]$ and $P_{0}=\mathrm{diag}\left[1000,1000,1000,1000,1000,1000\right]$ for both two Kalman filters. We set the amplitude ϑ=100 m in the noise driving matrix and the frequency τ=0.1 s in the process noise variance. The sampling time was also 0.2 s, and the total running time was 200 s. The estimated results using the different methods were recorded. The true and estimated target trajectories using EKF and CKF were drawn as well. The RMSEs between the estimated result and the true target states of the different methods are shown in Figure 26, Figure 27, Figure 28 and Figure 29.

The first example shows that the proposed CKF achieved satisfactory convergence performance when tracking a sinusoidal moving target. The CKF also had better estimation accuracy than that of the EKF in both position and velocity estimation. The EKF had to diverge in some of the Monte Carlo runs, resulting in target loss. In the second example, divergence did not happen in either method since the true target initial position became friendly to the EKF. However, the proposed CKF still had more accurate estimation results than those of the EKF. The effectiveness and superiority of the proposed CKF in mobile target tracking were verified.

## 7. Conclusions

This paper addressed moving-target detection and tracking problems in a 3D space with a proposed visual target tracking system only using a 2D camera. First, an improved optical flow method combined with a clustering algorithm was developed to accurately detect and extract a moving target from a noisy background. Second, a geometrical pinhole imaging algorithm was proposed to roughly and quickly estimate the target position. Third, the CKF algorithm was modified by directly using the camera pixel measurement for moving-target tracking in a 3D space. Simulation and experimental results demonstrated the effectiveness of the proposed systematic method. The work in this paper has great practical value, as it provides a solution for mobile-target detection and estimation using low-cost equipment.

In the future, we will further improve the accuracy of moving-target detection and estimation by considering the distortion problem of the camera lens. In addition, a pan–tilt–zoom serving system will be combined with the proposed system in this paper to further improve mobile-target tracking performance. 

## Figures and Tables

**Table 1:** Performance comparison of the improved PWC-Net on various datasets.

	Forecast	Sintel Clean		Sintel Final		KITTI 2012		KITTI 2015	
Network	Time	AEPE	AEPE	AEPE	AEPE	AEPE	AEPE	FL-All	FL-All
	(s)	Train	Test	Train	Test	Train	Test	Train	Test
PWC-Net	0.03	2.55	3.86	3.93	5.13	4.14	1.7	33.67%	9.60%
Improvement	0.03	2.33	2.77	3.72	4.38	3.21	1.1	23.58%	6.81%

**Table 2:** Experimental camera parameters.

Sensor specification	Advanced CMOS photosensitive chip, 1/2.7 inch
Pixel size	3 μm × 3 μm
Default speed	30 frames/s
Camera lens	Infrared, 60 degrees, no distortion
Signal to noise ratio	39 dB
Hardware	Industrial grade, 2 megapixels
Power	1 w
Working voltage	5 v
Output resolution	1920 × 1080
Interface	USB2.0, support UVC communication protocol
Focal length	6 mm

**Table 3:** Experimental results using the proposed method.

	Picture 1	Picture 2	Picture 3	Picture 4	Picture 5	Picture 6
Camera height	87.5 cm	87.5 cm	87.5 cm	87.5 cm	75.9 cm	75.9 cm
Shooting angle	45°	45°	45°	45°	30°	15°
Centroid position	(479,259)	(1,474,275)	(1,476,843)	(439,829)	(340,181)	(293,967)
Azimuth	−13.52°	14.41°	14.47°	−14.60°	−17.22°	−18.44°
Reference azimuth	−15.16°	20.5°	21.72°	−15.65°	−18.94°	−20.21°
Pitch	−8.00°	−7.55°	8.61°	8.22°	−10.18°	12.05°
Reference pitch	−9.85°	−9.54°	10.92°	10.79°	−11.69°	13.49°
Distance	149.5 cm	148.5 cm	112.1 cm	112.8 cm	234.1 cm	175.5 cm
Reference distance	157.4 cm	160.7 cm	113.4 cm	109.7 cm	241.3 cm	182.6 cm

## Data Availability

Not applicable.
